# Evaluation of the NO_x_ Reduction Performance of Mortars Containing Zeolite/Activated Red Clay Coated with a TiO_2_ Photocatalyst

**DOI:** 10.3390/ma17010080

**Published:** 2023-12-23

**Authors:** Bong-chul Joo, Hyeok-Jung Kim

**Affiliations:** 1 Department of Structural Research Modular Construction Research Cluster, Korea Institute of Civil Engineering & Building Technology, 283 Goyangdae-ro, Goyang-si 10223, Republic of Korea; bcjoo@kict.re.kr; 2 Industry Academic Cooperation Foundation, Hankyong National University, 327 Jungang-ro, Anseong-si 17579, Republic of Korea

**Keywords:** zeolite, NO_x_, activated red clay, TiO_2_

## Abstract

Globally, there is a growing concern about air pollution due to rapid industrialization and urbanization. Therefore, in this study, an experimental study was conducted to evaluate the performance of reducing nitrogen oxides, a precursor to fine dust, in mortars coated with a titanium dioxide (TiO_2_) photocatalyst, which has the effect of decomposing pollutants. In particular, in this study, zeolite and activated red clay were used as cement substitutes to improve the fine dust reduction performance of the TiO_2_ photocatalyst. A total of 14 different mixtures were designed, considering the substitution rates of zeolite and activated red clay (30%, 40%, and 50%) and the cement–fine aggregate ratio (1:2 and 1:3) as experimental variables. A TiO_2_ photocatalyst was employed in this study to evaluate the NO_x_ reduction performance. As zeolite and activated red clay were added, the compressive strength and flexural strength of the mortars decreased by 15% to 60%, while the absorption rate increased by 5% to 16%. The NO_x_ reduction efficiency of up to 67.4% was confirmed in the H50-3 specimen with the TiO_2_ catalyst. The NO_x_ reduction performance of mortars with the TiO_2_ photocatalyst sprayed on their surface improved as the substitution ratio of zeolite and activated red clay increased. Additionally, it was confirmed that the NO_x_ reduction effect of specimens using activated red clay was superior to those using zeolite. Therefore, through this study, it was confirmed that the NO_x_ reduction performance of the TiO_2_ photocatalyst can be improved when zeolite and activated red clay are used as cement substitutes.

## 1. Introduction

Globally, there is a growing concern about air pollution due to rapid industrialization and urbanization. One of the leading causes of air pollution is particulate matter (PM), which is typically categorized into general particulate matter (PM_10_), with a diameter of 10 μm or less, and fine particulate matter (PM_2.5_), with a diameter of 2.5 μm or less [1]. Due to their extremely small size, these particles can infiltrate the respiratory system, including the lungs and blood vessels, potentially leading to various respiratory diseases, such as asthma and bronchitis. Furthermore, precursor pollutants, like nitrogen oxides (NO_x_) and sulfur oxides (SO_x_), in the atmosphere, when reacting with moisture, ozone, and ammonia, can form particles that are notoriously difficult to remove [2]. Therefore, technologies that are capable of preemptively removing precursor pollutants are in demand for fine dust reduction. Photocatalysts are known to be effective in removing NO_x_, a precursor pollutant that constitutes a significant portion in urban and road environments and can contribute to the formation of fine dust particles [3,4]. Photocatalysts are catalytic materials that facilitate the decomposition reactions of precursor pollutants by irradiating them with light, such as ultraviolet radiation.

Among these photocatalysts, titanium dioxide (TiO_2_) is a material known for its relatively low cost, high chemical stability, and excellent decomposition performance. It finds applications in various fields, such as semiconductor materials, paints, cosmetics, and more. Particularly in densely populated urban areas, various sources, including vehicles, contribute to the emission of various NO_x_ pollutants [5]. However, due to the presence of tall buildings in urban centers, dispersing these emissions can be challenging. Therefore, the application of TiO_2_ on road surfaces and building exteriors can present a valuable strategy for mitigating fine dust pollution [6,7,8].

As a result, research on applying TiO_2_ photocatalysts to cement, a construction material, is being conducted by various researchers. For instance, Lackhoff et al. [9] conducted an experimental study where a TiO_2_ photocatalyst was added to cement mixtures. They reported that cement with an added TiO_2_ photocatalyst could decompose atmospheric pollutants on the surface of structures, making it applicable for the removal of pollutants generated under various environments. Furthermore, Jayapalan et al. [10] investigated the influence of a TiO_2_ photocatalyst on the initial hydration of cement. They considered variables such as the amount of TiO_2_ added to the cement and the particle size of TiO_2_. Their experiments revealed that an increase in the TiO_2_ addition rate accelerated the cement hydration rate, and smaller TiO_2_ particles enhanced the hydration reaction with cement [11,12]. Additionally, they suggested that the promoted hydration reaction due to the addition of TiO_2_ could be utilized to accelerate the strength development of cement pastes. Most of the studies conducted thus far have primarily been experimental studies aimed at applying TiO_2_ as a construction material. However, while cement-based construction materials incorporating TiO_2_ offer high durability and cost-effectiveness, addressing the fundamental issues associated with high energy consumption, carbon emissions, and dust emissions during cement production remains challenging.

To address these issues, Kwon et al. [13] conducted research on using eco-friendly construction materials such as zeolite and activated red clay as substitutes for cement. Zeolite is an aluminosilicate mineral with a three-dimensional mesh structure formed through the sharing of oxygen atoms between SiO_4_ and AlO_4_ tetrahedra [14,15,16]. This material has been known to contain numerous spaces occupied by water molecules and exchangeable cations [17,18,19]. The presence of exchangeable cations in zeolite allows for easy ion exchange, and their size and position influence various physicochemical properties [20,21]. The major components of zeolite include SiO_2_, Al_2_O_3_, and Fe_2_O_3_, which exhibit pozzolanic properties [22,23,24]. Additionally, this material has been valued for its excellent adsorption and catalytic properties, making it primarily used for deodorization and desiccation [25]. Perraki et al. [26] confirmed that incorporating zeolite into cement had little impact on its physical and mechanical properties, even when mixed at up to 20%. Activated red clay, on the other hand, shares similar components, such as SiO_2_ and Al_2_O_3_, with conventional concrete admixtures, making it exhibit pozzolanic characteristics [27]. Go et al. [28] reported that activated red clay reacts with Ca(OH)_2_ to produce 3CaO∙2SiO_2_∙3H_2_O (C-S-H) and 2CaO∙Al_2_O_3_∙SiO_2_∙8H_2_O (C-A-S-H) phases, making it suitable as a cement substitute. Furthermore, they highlighted the positive effects of activated red clay on strength development, odor removal, and heat retention [29].

It has been proven by several researchers that activated red clay and zeolite can be used as cement substitutes. As mentioned above, activated red clay and zeolite have excellent adsorption performances, so it has been expected that the application efficiency will increase when applying a TiO_2_ photocatalyst to mortars with activated red clay and zeolite added [30,31]. However, there are limited studies to evaluate the NO_x_ reduction performance of mortars containing zeolite and activated red clay with TiO_2_-coated photocatalysts. In order to apply new materials to the construction field, it is important to secure sufficient experimental results. Therefore, in this study, we experimentally assessed the physical properties and NO_x_ reduction performance of mortars using zeolite and activated red clay as cement substitutes.

## 2. Experimental and Numerical Procedures

### 2.1. Test Specimens

In this study, flexural specimens with dimensions of 40 mm in width, 40 mm in height, and 160 mm in length were prepared following ISO 4013 standards [32], and cubic compression strength specimens with a width of 40 mm were manufactured in accordance with ISO 679 standards [33]. Dust reduction experiments were conducted using specimens with dimensions of 50 mm in width, 100 mm in length, and 25 mm in height, as per ISO 22197-1 standards [34]. Table 1 presents the experimental variables and mixture designs for the specimens in which cement was substituted with zeolite and activated red clay for dust reduction. The water–binder ratio was kept constant at 25% for all mixtures, while the substitution materials (Z: zeolite; H: activated red clay), substitution ratios (0: 0%, 30: 30%, 40: 40%, and 50: 50%), and binder–aggregate ratios (1:2 and 1:3) were varied. For example, Z30-3 indicates that zeolite substituted 30% of the cement, and the binder–aggregate ratio was 1:3 in the mixture.

### 2.2. Used Materials

#### 2.2.1. Cement and Fine Aggregates

Table 2 illustrates the chemical composition of the ordinary Portland cement (OPC) type I that was used in this study. The specific surface area and density of the cement were 3265 cm^2^/g and 3.14 g/cm^3^, respectively. Table 3 presents the physical properties of the fine aggregates. The fine aggregates that were used had a specific gravity of 2.68 g/cm^3^, an absorption rate of 0.91%, and a fineness modulus of 2.81.

#### 2.2.2. Zeolite and Activated Red Clay

**Figure 1:**
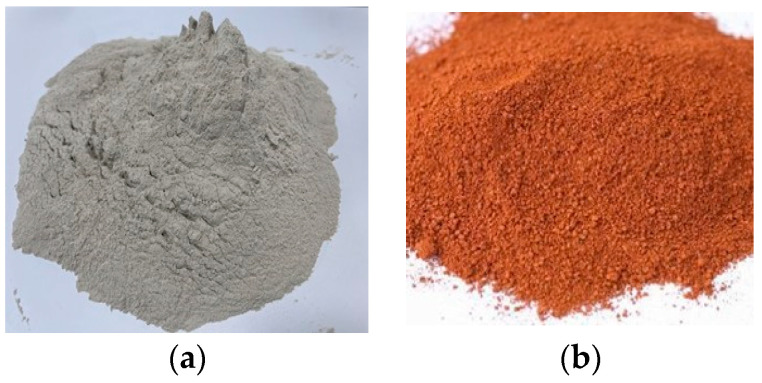
Zeolite and activated red clay powder. (**a**) Zeolite; (**b**) activated red clay.

Figure 1 and Table 4 depict the photographs and material properties of the zeolite(Paik Kwang Industruak Corporation, Gun-san, Republic of Korea) and activated red clay(Korea Ritek, Jin-ju, Republic of Korea) used in mortar production. The zeolite used in the mixture was in its natural state, with a specific surface area and density of 3000 cm^2^/g and 1.92 g/cm^3^, respectively. When clay is used in its natural state as a replacement for cement, it can lead to shrinkage and cracking issues [35]. In this study, activated red clay sintered at temperatures ranging from 800 °C to 850 °C was used to prevent these issues. This type of clay has a specific surface area and density of 3300 cm^2^/g and 2.50 g/cm^3^, respectively. Both zeolite and activated red clay have a porous structure, providing them with superior adsorption and catalytic performances compared to cement.

#### 2.2.3. Titanium Dioxide (TiO_2_) Photocatalyst

**Figure 2:**
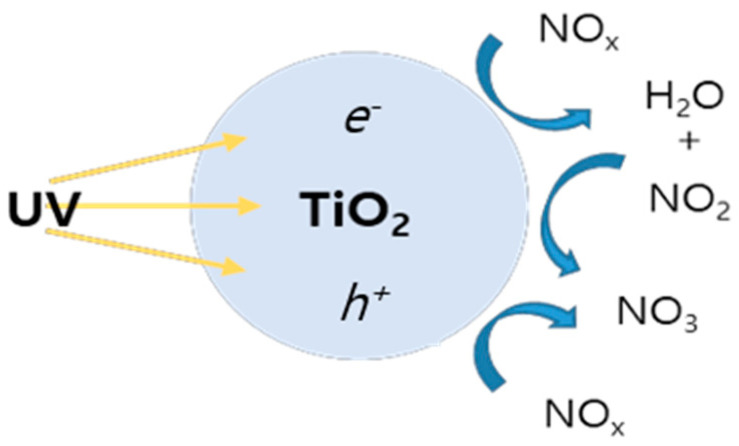
NO_x_ decomposition mechanism of the TiO_2_ photocatalyst.

The TiO_2_ photocatalyst(AERODISP^®^W740X, Evonik, Germany) used in this study possessed an anatase structure with a bandgap in the range from 3.0 eV to 3.2 eV. The NO_x_ reduction mechanism of this TiO_2_ photocatalyst operated as follows: When ultraviolet radiation with wavelengths below 387 nm is applied to the TiO_2_ photocatalyst, as depicted in Figure 2, electron–hole pairs (e− and h+) are generated in the conduction bands and valence bands, respectively. These pairs react with oxygen and moisture in the air, producing highly oxidative species, such as active oxygen (O_2_) and hydroxyl (OH) radicals, which can effectively decompose organic compounds. Subsequently, these generated active species participate in the decomposition of NO_x_ present in the atmosphere. Therefore, when ultraviolet rays are irradiated to the TiO_2_ photocatalyst applied to the mortar surface, O_2_ and OH are generated, and NO_x_ in the air is decomposed.

### 2.3. Test Methods

#### 2.3.1. Flexural and Compressive Strength Tests

**Figure 3:**
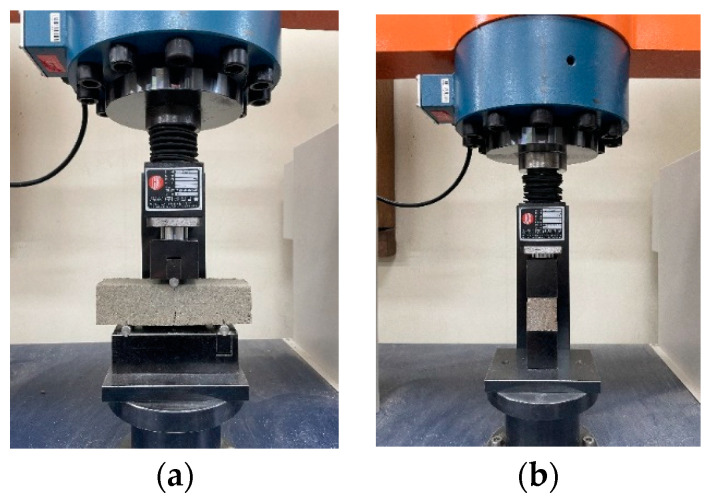
Test setup. (**a**) Flexural strength. (**b**) Compressive strength.

Flexural and compressive strength tests were conducted using a 300-kN-capacity universal testing machine (UTM; RT-M-045-D, RAMT, Seoul, Republic of Korea) in accordance with ISO 4013 and ISO 679 standards, respectively. Figure 3 illustrates the test setup of both the flexural and compressive strength experiments. These tests were performed at the curing ages of 3 days, 7 days, 28 days, 56 days, and 91 days. Flexural strength ($\sigma_{f}$) was calculated using Equation (1):

(1) $$\sigma_{f}=\frac{3Pl}{{2bd}^{2}}$$

where *P* represents the maximum load (N), *l* is the distance between support points (mm), and *b* and *d* denote the width (mm) and thickness (mm) of the specimen in the perpendicular direction to the supports, respectively.

#### 2.3.2. Absorption Test

The absorption test was conducted using flexural strength specimens that were cured for 28 days. For the absorption measurement, the specimens were submerged in clear water at 20 °C for 24 h, and the surface-dried mass ($m_{0}$) was measured after removing the surface moisture. Furthermore, the specimens in a surface-dried state were dried in an oven at 100–110 °C for 24 h, and the absolute dry mass ($m_{1}$) was measured. Equation (2) represents the formula that was used to calculate the absorption rate (*W_ab_*):

(2) $$W_{ab}(\%)=\frac{m_{0}-m_{1}}{m_{1}}\times 100$$

where *W_ab_* stands for the absorption rate (%), $m_{0}$ is the surface-dried mass (g), and $m_{1}$ is the absolute dry mass (g).

#### 2.3.3. NO_x_ Reduction Experiment

**Figure 4:**
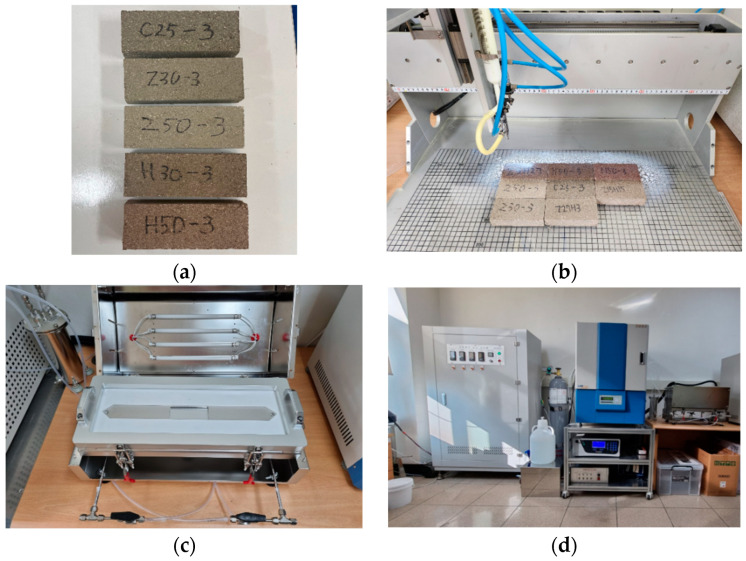
NO_x_ reduction experiment process. (**a**) Specimens; (**b**) TiO_2_ photocatalytic coating; (**c**) ISO standard chamber; and (**d**) NO_x_ reduction test setup.

Figure 4 illustrates the specimens and experimental setup prepared for the NO_x_ reduction experiment. The NO_x_ reduction experiment was conducted following ISO 22197-1 standards. For the NO_x_ reduction experiment, five specimens were prepared, each with a 30% and 50% substitution of cement with zeolite and activated red clay, respectively. To facilitate the NO_x_ reduction experiment, a TiO_2_ photocatalyst was coated on all the specimens using a spray coater, as depicted in Figure 4b. Additionally, an ISO 22197-1 standard chamber, as shown in Figure 4c, was utilized for this experiment. Figure 4d presents the test setup for the NO_x_ reduction experiment (NO_x_ analyser; Serinus^®^40, Ecotech). During the experiment, NO_x_ levels were continuously monitored for 7 h. A mixture of NO gas and air was used as a pollutant gas for the experiment with a NO concentration of 1 ppm. After placing the test specimen coated with the TiO_2_ photocatalyst in the ISO standard chamber, polluted gas was injected at a flow rate of 3 L/min. After stabilizing the sample with a constant flow rate of polluted gas in the chamber for 1 h, the test specimen was exposed to an ultraviolet (UV) lamp with a wavelength of 352 nm for 5 h. Then, the UV lamp was turned off, and the pollutant gas concentration was observed for 1 h to check whether the initial concentration was restored.

The efficiency of nitrogen oxide reduction was calculated using Equation (3):

(3) $$NER=\frac{{NO}_{x,stabilized}-{NO}_{x,equil}}{{NO}_{x,stabilized}}$$

where *NER* represents the NO_x_ reduction rate (%), *NO_x,stabilized_* denotes the stabilized NO_x_ concentration, and *NO_x,equil_* represents the NO_x_ concentration at equilibrium during the photolytic reaction of NO_x_.

## 3. Results and Discussion

### 3.1. Flexural Strength Test Results

**Figure 5:**
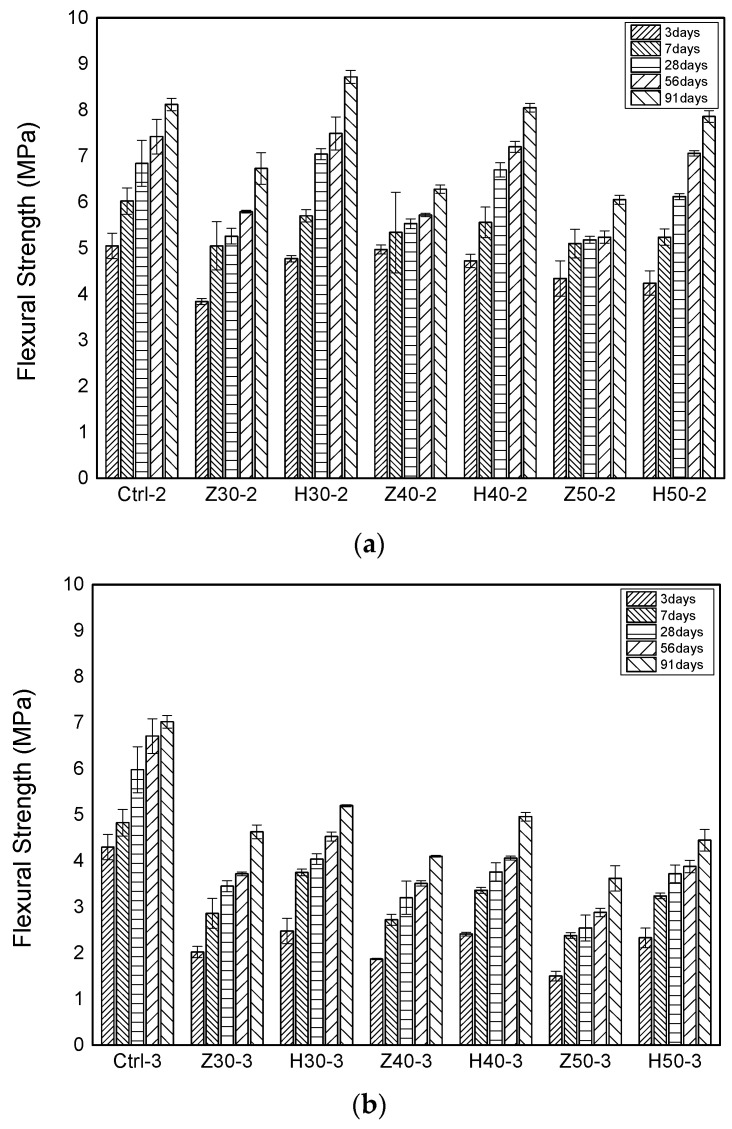
Flexural strength. (**a**) Specimens with a fine aggregate ratio of 1:2; and (**b**) specimens with a fine aggregate ratio of 1:3.

Figure 5 presents the flexural strength test results according to the curing age. For the reference specimens C25-2 and C25-3, which did not incorporate zeolite or activated red clay, their flexural strengths at 7 days were 6.02 MPa and 4.83 MPa, respectively, and at 28 days, they were 6.84 MPa and 5.98 MPa, respectively. Subsequently, at 91 days, the flexural strengths of C25-2 and C25-3 increased by an average of 18.7% and 17.4%, respectively, compared to their 28-day strengths. For the specimens with the zeolite substitution, their flexural strengths at 28 days decreased by 31.2%, 31.7%, and 38.9% as their substitution ratios were increased to 30%, 40%, and 50%, respectively, compared to the reference specimens. Furthermore, for the specimens with the activated red clay substitution, their flexural strengths at 28 days decreased by 14.9%, 19.7%, and 23.8%, respectively. An overall trend that was observed was that as the substitution ratio of zeolite and activated red clay increased, the strength decreased compared to the reference specimens. Notably, for the Z50-3 specimen with a 50% zeolite substitution, its strength decreased by 48% compared to the reference specimens at 91 days. This was attributed to inadequate strength development and insufficient pozzolanic reactions occurring as the substitution ratio of zeolite and activated red clay for cement increased. Notably, zeolite had a more pronounced adverse effect on flexural strength compared to activated red clay.

### 3.2. Compressive Strength Test Results

**Figure 6:**
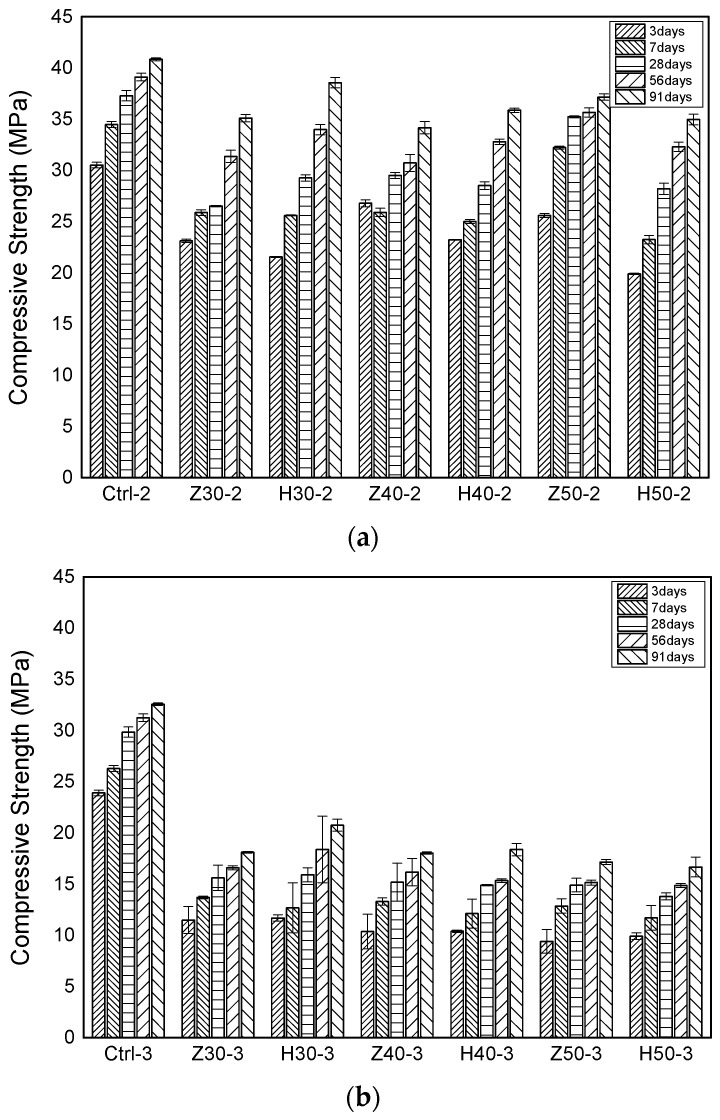
Compressive strength. (**a**) Compressive strength of a fine aggregate ratio of 1:2; and (**b**) compressive strength of a fine aggregate ratio of 1:3.

Figure 6 presents the results of the compressive strength tests under various curing ages. For the reference specimens C25-2 and C25-3, which did not incorporate zeolite or activated red clay, their compressive strengths at 28 days were 37.29 MPa and 29.84 MPa, respectively. In contrast, the specimens with the zeolite and activated red clay substitutions exhibited a reduction in their strengths ranging from 5.52% to 50.1% compared to their 28-day reference specimens. Among the specimens with a 1:2 binder-to-aggregate ratio, their strength reduction was below 28.9% at 28 days. However, for specimens with a 1:3 binder-to-aggregate ratio, all of them exhibited reductions close to 50%. Furthermore, at 91 days, the compressive strengths of specimens with zeolite and activated red clay substitutions were highest for the H30-2 and H30-3 specimens, measuring 38.56 MPa and 20.75 MPa, respectively. In both the 1:2 and 1:3 binder-to-aggregate ratios, specimens with a 30% activated red clay substitution exhibited the highest strengths. However, all the specimens with the zeolite and activated red clay substitutions showed lower compressive strengths compared to their reference specimens. The phenomenon of compressive strength decreasing as the amount of pozzolanic material substituted increased appeared similarly in other researchers’ studies. Vejmelkova et al. [36] reported that the compressive strength of their specimen with a 40% zeolite substitution decreased by about 45.6% compared to the compressive strength of their specimen that only contained cement. Additionally, they reported that the rate of increase in the compressive strength of their specimens that were mixed with zeolite increased faster than that of their specimens that only used cement, and they claimed that this was due to a pozzolanic reaction. Lin et al. [37] reported that the 28-day compressive strength of a specimen with a 30% replacement of activated red clay decreased by approximately 15.3% compared to a specimen that only used cement, and they claimed that this was due to a decrease in the cement content in their paste.

### 3.3. Absorption Rate Test Results

Table 5 presents the absorption rate test results measured at 28 days of curing. Among the specimens, H30-2, which had a zeolite substitution of 30% with a 1:2 binder-to-aggregate ratio, exhibited the lowest absorption rate at 7.37%. The specimen Z50-3, which had a 50% zeolite substitution with a 1:3 binder-to-aggregate ratio, showed the highest absorption rate at 9.22%. Specimens with a 1:2 binder-to-aggregate ratio exhibited similar absorption rates when zeolite or activated red clay was substituted, ranging from 7.37% to 7.41%. However, specimens with a 1:3 binder-to-aggregate ratio showed an increase in their absorption rate with an increase in zeolite substitution. Therefore, in this experiment, when using the same substitution ratio, the absorption rate of activated red clay was found to be lower than that of zeolite. Additionally, increasing the substitution ratio of activated red clay did not result in an increase in the absorption rate.

### 3.4. NO_x_ Reduction Performance

In order to evaluate the nitrogen oxide removal performance of the photocatalyst according to the type and content of the cement admixture, a mortar containing activated red clay and zeolite was produced, and after coating with the TiO_2_ photocatalyst, a nitrogen oxide removal experiment was performed according to the ISO 22197-1: 2016 test method.

**Figure 7:**
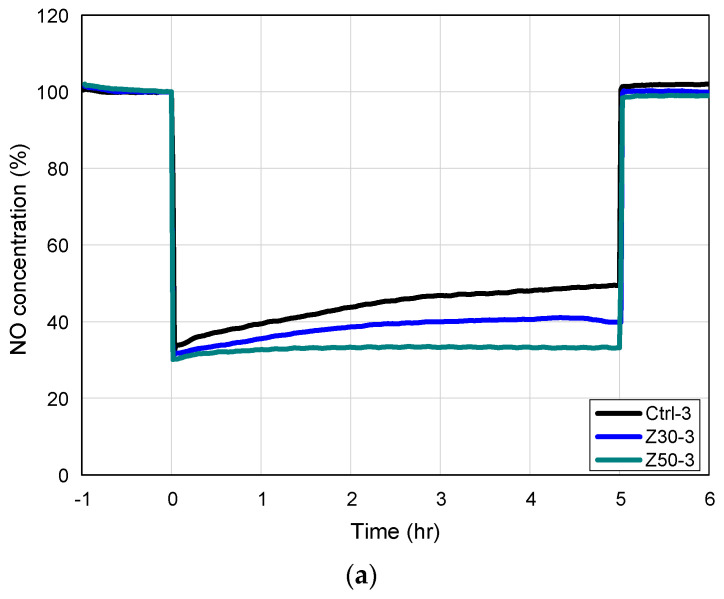

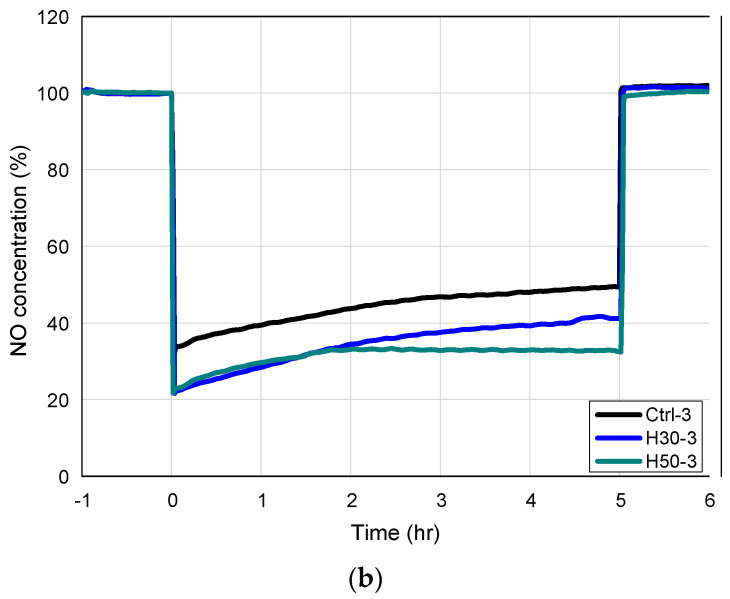
NO_x_ reduction. (**a**) The NO_x_ reduction test with zeolite; and (**b**) the NO_x_ reduction test with activated red clay.

The results of evaluating the NO_x_ reduction performance according to the type and content of admixture are shown in Figure 7, and the average NO_x_ reduction efficiency values of each specimen are summarized in Table 6. In all the specimens with a photocatalytic coating, it was confirmed that the concentration of input pollutant gas was maintained at 1 ppm under a dark atmosphere without photocatalytic reactions for the first 1 h. After turning on the UV lamp, the concentration of NO gas rapidly decreased due to the photocatalytic reaction and gradually reached an equilibrium state. Then, turning off the UV ramp deactivated the photocatalytic reaction and restored it to its initial concentration [38]. In the case of the standard specimen Ctrl-3, its NO_x_ reduction efficiency was approximately 54.2% due to the NO_x_ decomposition using the TiO_2_ photocatalyst. In comparison, the NO_x_ reduction efficiencies of Z30-3, H30-3, Z50-3, and H50-3, which replaced part of the cement (Ctrl-3) with admixtures, exhibited higher reduction performances, ranging from 6.4% to 13.2%. Moreover, the NO_x_ reduction efficiency was found to increase as the zeolite and activated red clay contents were increased. Notably, specimen H50-3, which had a 50% activated red clay substitution, had the best NO reduction rate of 67.4%. This was attributed to its high contents of zeolite and activated red clay, which have abundant pores and excellent adsorption capabilities, facilitating the effective adsorption of the TiO_2_ photocatalyst. Ultimately, it was determined that using an admixture of zeolite and activated red clay can be used as a construction material with a nitrogen oxide removal function.

## 4. Conclusions

In this study, NO_x_ reduction experiments were conducted on mortars incorporating zeolite and activated red clay, leading to the following conclusions:

The flexural strengths of mortars with zeolite and activated red clay substitutions showed that the flexural strength of activated red clay was superior to that of zeolite. As the substitution ratio of zeolite and activated red clay increased, the reduction in strength compared to conventional cement concrete also increased. It was observed that mortars with zeolite substitutions exhibited a 10% to 20% lower flexural strength compared to those with activated red clay substitutions.

Compressive strength exhibited a similar trend to flexural strength. The substitution of zeolite and activated red clay resulted in a reduction in compressive strength ranging from 2.29 MPa to 15.89 MPa compared to the standard specimens. Moreover, specimens with a cement–fine aggregate ratio of 1:3 showed a greater reduction in their strength.

The absorption rate increased as the substitution ratio of zeolite and activated red clay increased in absorption experiments. Zeolite exhibited approximately 5.13% to 16.27% higher absorption rates compared to activated red clay.

The NO_x_ reduction performance was improved by replacing cement with zeolite and activated red clay by up to 12.6% and 13.2%, respectively. Furthermore, activated red clay demonstrated better NO_x_ reduction effects compared to those achieved with zeolite. It will be necessary to identify its cause through future research, such as micropore analyses.

Through this study, it was confirmed that the NO_x_ reduction efficiency of a TiO_2_ photocatalyst can be increased by adding zeolite and activated red clay. Therefore, it is expected that using zeolite and activated red clay as a cement substitute and applying TiO_2_ photocatalysts to construction materials that use mortar, such as interlocking blocks and sidewalk blocks, will be effective in reducing roadside NO_x_ in the future.

## Figures and Tables

**Table 1:** Mixing design.

Type	Unit Weight (kg/m^3^)				
	Water	Binder			Sand
		Cement	Zeolite	Activated Red Clay	
C25-2	300	1200	-	-	2400
Z30-2		840	360	-	
H30-2			-	360	
Z40-2		720	480	-	
H40-2			-	480	
Z50-2		600	600	-	
H50-2			-	600	
C25-3		1200	-	-	3600
Z30-3		840	360	-	
H30-3			-	360	
Z40-3		720	480	-	
H40-3			-	480	
Z50-3		600	600	-	
H50-3			-	600	

**Table 2:** Chemical composition of used cement.

SiO_2_ (%)	Al_2_O_3_ (%)	Fe_2_O_3_ (%)	CaO (%)	MgO (%)	SO_3_ (%)
21.74	5.00	3.17	62.79	2.97	1.67

**Table 3:** Physical properties of fine aggregates.

Type	Specific Gravity (g/cm^3^)	Fineness Modulus	Absorption Rate (%)
River sand	2.68	2.81	0.91

**Table 4:** Physical properties of zeolite.

	SiO_2_ (%)	Al_2_O_3_ (%)	Fe_2_O_3_ (%)	CaO (%)	MgO (%)	K_2_O (%)
Zeolite	68.9	16.4	5.3	2.6	1.0	3.7
Activated red clay	43.0	25.9	10.8	7.2	1.6	0.8

**Table 5:** Absorption rate test results.

Type	Absorption Rate (%)	Type	Absorption Rate (%)
Ctrl-2	7.18	Ctrl-3	7.33
Z30-2	7.98	Z30-3	8.69
H30-2	7.37	H30-3	7.87
Z40-2	7.79	Z40-3	8.80
H40-2	7.41	H40-3	7.80
Z50-2	8.12	Z50-3	9.22
H50-2	7.39	H50-3	7.93

**Table 6:** NO_x_ reduction results.

Type	Efficiency of NO_x_ Reduction (%)
Ctrl-3	54.2
Z30-3	60.6
H30-3	63.6
Z50-3	66.8
H50-3	67.4

## Data Availability

Data are contained within the article.
